# The Plea of Humanity in Behalf of Medical Education

**Published:** 1849

**Authors:** 


### ARTICLE VI.

*The Plea of Humanity in behalf of Medical Education:*— The Annual Address delivered before the New York State Medical Society, and Members of the Legislature, at the Capitol, February 6, 1849. By ALEXANDER H. STEVENS, M. D., President of the Socity, of the American Medical Association, and of the College of Physicians and Surgeons of New York, Member of the Philosophical Society of Philadelphia, etc. Published by the Society.

*Anniversary Discourse before the New York Academy of Medicine, November*, 8, 1848. By JAMES R. MANLEY, M. D. Published by order of the Academy,

*Valedictory Address to the Medical Class of Transylvania University.* By E. L. DUDLEY, M. D., Professor of General and Pathological Anatomy and Physiology. Published by the Class.

The first of these is from one of the sages of the profession whose teachings are sound, and whose style and matter are always of the most interesting character. It is a document of the highest order of interest, containing a large amount of statistical information in reference to the utility and influence of our profession, when rightly educated and properly skilled, upon the health, prosperity, and happiness of our race.

After showing the large indebtedness of the world to our profession for the great advancement of the arts and sciences, and for the impulse given by it to the cause of benevolence, in its various features religious, moral, and humane, the author says:

But the relations of medicine to education, to social happiness, agriculture, virtue, science, temperance, and philanthropy, however important, are of far less value than its legitimate object, the restoration to, and the preservation of health. From the cradle to the grave, aye, even from a period antecedent to its birth, every individual enjoys the advantages of medical science. Almost every child receives the blessing of vaccination, and is thereby protected from a loathsome and often fatal pestilence. A very large portion of the community are placed by disease or injury in a condition in which their usefulness or their lives are preserved solely by medical skill.

Throughout the civilized world, the duration of human life has increased, and is steadily increasing with the advancement and diffusion of medical science and medical art.

In the city of Geneva, in the 16th century, 1 individual in 25 died annually. For the 18th century, 1 in 34; at the present time, 1 in 46.* With us the mortality is greater. I estimate it at 1 in 40, the proportion of childhood being larger and childhood being the period of the greatest mortality. In the British navy among adults, none of whom are very aged, the mortality is only 1 in 100. Seventy years ago the mortality was 1 in every 10. In 1808, 1 in 30; 1836, 13·8, among one thousand—a diminution to less than a seventh of the rate in 1770. In the American army, with a corps of medical officers, not excelled by that of any other country, the mortality is little over 1 in 300 per annum. In London the mortality in the middle of the last century was 1 in 32. In the year 1838, the mortality was 1 in 36. I quote from the annual report of the Register General. Within the last twenty years the mortality of Russia has been 1 in 27. Prussia, 1 in 36; France, 1 in 39·07; Holland, 1 in 39; Belgium, 1 in 43·01; England, 1 in 53·07; Sicily, 1 in 32; Greece, 1 in 30; Philadelphia, 1 in 42·03; Boston, 1 in 45; New York, 1 in 37·83·† the imigrants have made our mortality greater than that of our sister cities; in other respects it has diminished with the advance of medical science. These statistical statements might be multiplied at great length; but enough have been given to show conclusively the prodigious extent to which human life has been lengthened with the advance and diffusion of medical science beyond its duration in former periods, and beyond its present duration in the less enlightened countries of Europe.

* Annales d' Hygeine Publique.

† Bell on Longevity, N. Y. Hosp. Library.

Again, in reference to the correct management of hospitals he says:

Until within the last two years the New York Alms-house, with its hospital, has been under political management. The profession had no part in directing or advising in regard to the choice of its physicians. The mortality during a period of 20 years was more than 20 per cent. per annum,

Two years since a new organization was made, and the whole establishment was placed under the control of one resident physician and an efficient corps of unpaid physicians and surgeons. During these two years, the mortality has been reduced to about 12 per cent. per annum, which is about 1 per cent. above that of the Pennsylvania Hospital, the Massachusetts General Hospital, and the New York Hospital; all which institutions have long been manged well.

A very large edition of this address has been published by the Society and the legislature of New York. This is encouraging, as it is only the diffusion of correct information amongst the people, such as this address contains, that is necessary to lead the community at large to appreciate scientific attainments and skill in our profession.

The proposition made by the eminent author for making medical education free, to have colleges supported by the state governments, furnishes matter for much argument pro and con. We have no doubt the plan would be advocated by many were there a reasonable prospect of its adoption.

The address of Dr. Manly shows the author to belong to the weeping school of philosophers, as he finds the profession wrong and growing rapidly worse. Too many medical schools, and incompetent teachers are among the causes of the degradation that has now come upon us. That there is room for great improvement we have no doubt, but we do not give up all hope, (although our staff and wig may be gone,) that our dignity may be so far restored that we will command the respect due our moral and intellectual worth. We know of no place where an intelligent and upright physician who pursues his profession faithfully and steadily, will not occupy a station of respectability and influence in society. Quackery has always existed, and, for aught we can see, is likely to continue; but that it is to supplant the medical profession we have no fears.

The recommendation that physiology be made a branch of popular education is good, and as a means of qualifying communities to judge of the claims of scientific physicians to their confidence may be correct, but we opine if it be the only hope of a restoration of our professional dignity, many generations of us will yet go down to the grave undignified.

Prof. Dudley's Address is beautifully written, and dwells upon the bright side of the character of our profession, pointing out the pleasures attending its practice.

It is an eloquent appeal to the young physician in behalf of a pure morality and a lofty aim in his career through life, alike creditable to the author's head and heart. E.

